# Imaging biomarkers in Parkinson’s disease and Parkinsonian syndromes: current and emerging concepts

**DOI:** 10.1186/s40035-017-0076-6

**Published:** 2017-03-28

**Authors:** Usman Saeed, Jordana Compagnone, Richard I. Aviv, Antonio P. Strafella, Sandra E. Black, Anthony E. Lang, Mario Masellis

**Affiliations:** 1 Institute of Medical Science, Faculty of Medicine, University of Toronto, Toronto, Canada; 2 LC Campbell Cognitive Neurology Research Unit, Sunnybrook Research Institute, Toronto, Canada; 3 Department of Medical Imaging, University of Toronto and Division of Neuroradiology, Sunnybrook Health Sciences Centre, Toronto, Canada; 4 Research Imaging Centre, Centre for Addiction and Mental Health, Toronto, Canada; 5 Division of Brain, Imaging & Behaviour - Systems Neuroscience, Toronto Western Hospital, Toronto, Canada; 6 Division of Neurology, Department of Medicine, University of Toronto, Toronto, Canada; 7 Heart & Stroke Foundation Canadian Partnership for Stroke Recovery, Sunnybrook Health Sciences Centre, Toronto, Canada; 8 Movement Disorders Centre, Toronto Western Hospital, Toronto, Canada; 9 Edmond J. Safra Program in Parkinson’s Disease, University Health Network, Toronto, Canada; 10 Cognitive & Movement Disorders Clinic, Sunnybrook Health Sciences Centre, 2075 Bayview Ave., Room A4-55, Toronto, Ontario M4N 3 M5 Canada

**Keywords:** Biomarkers, Parkinson’s disease, Atypical parkinsonian syndrome, MRI, PET, SPECT, Diffusion tensor imaging, Molecular imaging, Myocardial scintigraphy, Transcranial sonography

## Abstract

Two centuries ago in 1817, James Parkinson provided the first medical description of Parkinson’s disease, later refined by Jean-Martin Charcot in the mid-to-late 19th century to include the atypical parkinsonian variants (also termed, Parkinson-plus syndromes). Today, Parkinson’s disease represents the second most common neurodegenerative disorder with an estimated global prevalence of over 10 million. Conversely, atypical parkinsonian syndromes encompass a group of relatively heterogeneous disorders that may share some clinical features with Parkinson’s disease, but are uncommon distinct clinicopathological diseases. Decades of scientific advancements have vastly improved our understanding of these disorders, including improvements in *in vivo* imaging for biomarker identification. Multimodal imaging for the visualization of structural and functional brain changes is especially important, as it allows a ‘window’ into the underlying pathophysiological abnormalities. In this article, we first present an overview of the cardinal clinical and neuropathological features of, 1) synucleinopathies: Parkinson’s disease and other Lewy body spectrum disorders, as well as multiple system atrophy, and 2) tauopathies: progressive supranuclear palsy, and corticobasal degeneration. A comprehensive presentation of well-established and emerging imaging biomarkers for each disorder are then discussed. Biomarkers for the following imaging modalities are reviewed: 1) structural magnetic resonance imaging (MRI) using T1, T2, and susceptibility-weighted sequences for volumetric and voxel-based morphometric analyses, as well as MRI derived visual signatures, 2) diffusion tensor MRI for the assessment of white matter tract injury and microstructural integrity, 3) proton magnetic resonance spectroscopy for quantifying proton-containing brain metabolites, 4) single photon emission computed tomography for the evaluation of nigrostriatal integrity (as assessed by presynaptic dopamine transporters and postsynaptic dopamine D2 receptors), and cerebral perfusion, 5) positron emission tomography for gauging nigrostriatal functions, glucose metabolism, amyloid and tau molecular imaging, as well as neuroinflammation, 6) myocardial scintigraphy for dysautonomia, and 7) transcranial sonography for measuring substantia nigra and lentiform nucleus echogenicity. Imaging biomarkers, using the ‘multimodal approach’, may aid in making early, accurate and objective diagnostic decisions, highlight neuroanatomical and pathophysiological mechanisms, as well as assist in evaluating disease progression and therapeutic responses to drugs in clinical trials.

## Background

With an estimated global prevalence of more than 10 million cases [1], Parkinson’s disease (PD) represents the second most common neurodegenerative disorder after Alzheimer’s disease (AD), associated with momentous socioeconomic burden and immeasurable human suffering. The first medical description of PD was provided by James Parkinson nearly two centuries ago. Since then, the precise conceptualization of this disease has evolved considerably [2]. In the mid-to-late 19th century, Jean-Martin Charcot curiously illustrated the atypical parkinsonian variants and likened the unusually extended extremities of one of his patients to ‘rigid bars’ [2]. Decades of scientific advances in diagnostics, neuroimaging and clinicopathological correlations have permitted a better understanding of PD and related atypical parkinsonian syndromes (PS) (also termed Parkinson-plus syndromes).

PD is characterized by the death of dopaminergic neurons within the substantia nigra pars compacta (SNpc) due to intraneuronal aggregation of α-synuclein in the form of Lewy bodies and Lewy neurites in the majority of cases [3]. The resultant dopaminergic denervation in the basal ganglia combined with dysfunction in non-dopaminergic systems due to more widespread neurodegeneration leads to classical motor and non-motor symptoms. Motor manifestations of PD include bradykinesia, rigidity, resting tremor, and postural and gait disturbances; whereas, non-motor features may include depression, olfactory and autonomic dysfunction, sleep disorders, psychiatric symptoms, pain, fatigue and cognitive impairment [3]. Conversely, atypical PS encompass a group of relatively heterogeneous disorders that may share some clinical features with PD, but are distinct clinicopathological entities.

Neurodegenerative diseases are now classified on the basis of the predominant protein aggregates that characterize the neuropathology. These proteins are believed to play a critical role in disease pathogenesis. In the case of parkinsonian disorders, the predominant underlying neuropathologies include: 1) α-synucleinopathies, such as the full clinical spectrum of PD with and without cognitive impairment/dementia, dementia with Lewy bodies (DLB), and multiple system atrophy (MSA); and 2) tauopathies, including progressive supranuclear palsy (PSP) and corticobasal degeneration (CBD). A small proportion of cases, e.g. presenting with a corticobasal syndrome, are classified as TDP-43opathies (pathology of transactive response DNA binding protein of 43 kDa). Overlap in symptomatology, clinical heterogeneity in disease presentation and progression, and variability in response to dopaminergic medications can make the differential diagnosis of parkinsonian disorders challenging at times, especially at early disease stages. Accurate and prompt diagnosis is vital to accommodate differential prognostic and disease management approaches, and to assess the efficacy of experimental therapeutic interventions in clinical trials. Imaging plays a pivotal role in this regard by providing an *in vivo* opportunity to visualize the neuroanatomical and functional signatures of these disorders, as well as identifying disease-specific biomarkers of the underlying neurodegenerative processes. These biomarkers have the potential to eventually serve as reliable neuropathologic indicators to improve the sensitivity and specificity of clinical diagnoses.

This review will present a brief overview of the prominent clinical and neuropathological features of parkinsonian disorders, followed by a comprehensive presentation of well-established and promising imaging biomarkers with emphasis on their distinguishing characteristics in PD and atypical PS.

## Methods

The literature was comprehensively reviewed via the PubMed database using the following disease-specific keywords: ‘Parkinson*’, ‘Lewy*’, ‘multiple system atrophy’, ‘corticobasal degeneration’, ‘progressive supranuclear palsy’; − combined with one of the modality-specific terms: ‘magnetic resonance imaging’, ‘positron emission tomography’, ‘single-photon emission computed tomography’, ‘diffusion tensor’, ‘proton spectroscopy’, ‘myocardial scintigraphy’, and ‘transcranial sonography’. Acronyms, e.g. ‘MRI’ for ‘magnetic resonance imaging’, were entered as appropriate. Articles were restricted to those: 1) in English, and 2) published between January 1, 1995 and February 29, 2016. All identified abstracts were screened for relevance and the most pertinent articles were reviewed in full, with further examination of the corresponding reference lists, which became the foundation for this review.

## Clinical and neuropathological features

### α-synucleinopathies – Lewy body spectrum disorders and multiple system atrophy

Lewy body spectrum disorders (LBD) include a clinical spectrum of closely-related α-synucleinopathies that share clinical characteristics of levodopa-responsive parkinsonism, cognitive impairment, fluctuations in attention and alertness, and visual hallucinations to varying degrees. These disorders include PD with or without mild cognitive impairment (MCI), Parkinson’s disease dementia (PDD) and dementia with Lewy bodies (DLB). While motor symptoms predominate at early PD stages, cognitive dysfunction generally emerges later. Until recently, the onset of parkinsonism relative to dementia manifestation was used as an arbitrary criterion to clinically distinguish PDD and DLB using a ‘one-year rule’: dementia onset within 12-months of or contemporarily with motor dysfunction qualified as DLB, whereas parkinsonism had to precede dementia by at least one-year in PDD [4]. However, recent diagnostic criteria for PD developed by the International Parkinson and Movement Disorder Society propose eliminating this arbitrary one-year rule. All patients fulfilling diagnostic criteria for PD are diagnosed as such, independent of when dementia develops. Predominant brainstem Lewy pathology is seen in PD, whereas more diffuse Lewy pathology involving the brainstem, limbic and neocortical regions is typical of DLB and PDD. Concurrent AD pathology (amyloid-beta [Aβ] plaques and neurofibrillary tangles) frequently co-exists in DLB and may even be seen in PDD, thus contributing to substantial clinical heterogeneity in these disorders [4, 5].

Multiple system atrophy (MSA) represents an adult-onset, heterogeneous neurodegenerative disease with progressive autonomic and/or cerebellar dysfunction, and encompasses three disorders that were formerly considered distinct clinicopathological conditions: Shy-Drager syndrome, olivopontocerebellar atrophy, and striatonigral degeneration [6]. Common clinical symptoms of MSA include motor features, such as parkinsonism, cerebellar ataxia and postural abnormalities; and non-motor features secondary to autonomic failure involving multiple physiological systems – cardiovascular and urogenital being the most frequently affected [6]. Broadly, MSA is subgrouped into the parkinsonian subtype (MSA-*P*) if parkinsonism is the predominant presentation; or cerebellar subtype (MSA-*C*) with characteristic cerebellar symptomatology. Histopathological examination reveals oligodendroglial cytoplasmic inclusions (Papp-Lantos bodies) housing misfolded α-synuclein protein with varying degrees of degeneration in olivopontocerebellar and striatonigral regions [6].

### Tauopathies – progressive supranuclear palsy and corticobasal degeneration

Formally described in 1964 by Steele, Richardson and Olszewski, PSP is a progressive neurodegenerative disease associated with axial rigidity, bradykinesia, postural instability, vertical supranuclear gaze palsy, speech and swallowing dysfunction, as well as fronto-executive cognitive and behavioural manifestations [7, 8]. Gait impairment typically progresses at an accelerated rate in PSP relative to PD, with early falls as a prominent feature. Several variants have been identified that challenge the classical clinicopathological characterization of the PSP syndrome. For example, unlike the classical syndrome (now referred to as Richardson syndrome or PSP-*R*), the PSP-parkinsonism variant (PSP-*P*) exhibits more conspicuous limb rigidity with bradykinesia and/or tremor with moderate levodopa response in a proportion of patients, without early ocular or postural disturbances [8]. Vertical gaze palsy is an important diagnostic feature of PSP, although it may not be evident at early disease stages [8]. Histopathologically, evidence of neurofibrillary tangles composed of misfolded 4-repeat tau protein, neuropil threads and star-shaped tufted astrocytes are seen, mainly in the basal ganglia, brainstem and diencephalon [7, 8].

The classical syndrome associated with CBD pathology involves the combination of basal ganglionic and cortical features. Strikingly asymmetric limb rigidity, dystonia, and bradykinesia are basal ganglionic features, whereas typical cortical features include limb apraxia, aphasia, alien limb phenomenon, stimulus-sensitive myoclonus as well as other cognitive and behavioural impairments [9]. Neuropathological findings reveal abnormal accumulation of hyperphosphorylated 4-repeat tau in the form of swollen, achromatic (i.e., ballooned) neurons, and in glial cells as astrocytic plaques [9]. Diagnostic accuracy of CBD is modest due to extensive neuropathological heterogeneity and merely 25–56% of cases are correctly diagnosed antemortem [9, 10]. This represents a foremost obstacle in research studies, where antemortem diagnosis is confounded by a host of other underlying pathologies with overlapping features, including AD, PSP and other tau-positive and tau-negative (largely TDP-43 positive) forms of frontotemporal lobar degeneration (FTD) [11]. A broader term, corticobasal syndrome (CBS), has thus been suggested to describe clinical characterization of this disorder without histopathological confirmation.

## Magnetic resonance imaging in Parkinsonian disorders

### Structural magnetic resonance imaging

Magnetic resonance imaging (MRI) takes advantage of abundant hydrogen atoms and strong magnetic fields to image brain tissues non-invasively. Conventional structural MRI uses distinct pulse sequences to obtain T1-weighted (T1), T2-weighted (T2), proton-density weighted, fluid-attenuated inversion recovery (FLAIR) and/or susceptibility-weighted (SW) scans. SW imaging is sensitive to magnetic inhomogeneity effects, particularly due to iron accumulation, hemorrhages, and/or slow venous blood flow, allowing for an enhanced tissue contrast. These images can be analyzed selectively or in combination to obtain volumes of brain structures, regional cortical thickness, and to identify regional tissue abnormalities. Such structural profiles, including patterns and rates of atrophy, are important areas of research from a biomarker viewpoint.

#### Structural MRI profiles of Parkinson’s disease

Structural brain changes tend to be subtle in early PD and may not be apparent on conventional MRI. Voxel-based morphometry (VBM) studies have identified reduced gray matter (GM) volumes compared to controls in the frontal lobe [12], right hippocampus, and left anterior cingulate and superior temporal gyri [13]. Pitcher et al. detected an 11% and 8% reduction in caudate and putaminal volumes respectively in PD patients relative to controls [14]. Tinaz and colleagues applied MRI coupled with an automated surface reconstruction method and reported cortical thinning in the orbitofrontal, ventrolateral prefrontal, and occipitoparietal cortical regions in PD subjects, along with striatal volumetric reductions subcortically [15]. However, normal striatal volumes have also been reported in PD, whereas brainstem volumes were found to be significantly reduced in MSA and PSP [16]. In a recent VBM study, Chen et al. found significant volumetric loss in the olfactory bulb and tracts of PD patients, versus MSA and controls, and the global olfactory bulb volume inversely correlated with PD duration [17].

As PD is associated with substantia nigra (SN) pathology, several investigations have aimed to identify SN volumetric differences on MRI, although with varying results. Some studies noted no volumetric differences in SN compared to controls [18, 19], while others identified a decrease [20] and even an increase in SN volume [21]. Minati et al. reported smaller SN with a characteristic lateral-to-medial loss [20], whereas Péran and colleagues found no volumetric changes, but identified an increased R2* transverse relaxation rate in SN [19]. More recent research using ultra-high-field MRI allowed for finer structural resolution of SN and helped to clarify volumetric discrepancies. At 7 Tesla (T), Cho et al. described ‘smudging’ or loss of the fine boundaries between SN and crus cerebri, which appeared relatively ‘serrated’ in PD patients [22]. Similarly, the identification of a three-layered anatomical organization of SN became evident on SW 7 T-MRI in normal controls, which was less apparent or unidentifiable in PD subjects. This SW-derived architectural change of SN at 7T allowed excellent discrimination of PD versus normal controls (sensitivity 100%, specificity 96.2%) [23]. Such morphological alterations, presumably due to degeneration and iron accumulation in SNpc, may produce expanded hypointense regions on MRI and explain the increased SN volumes reported in some studies at lower resolutions. These changes also align with longitudinal reports of increased R2* relaxation rates of SN in PD (~10.2% in pars compacta; 8.1% in pars reticula over a three-year period) [24], which can result from ferritin-induced field inhomogeneities and are shown to correlate with worsening motor symptoms. In a recent 36-month longitudinal study, a greater change in R2* relaxation rates in SNpc was found among patients destined to develop freezing of gait early in PD [25]. This study, however, was limited by a small sample size (*n* = 19), requiring validation in a larger cohort.

Visualization of early changes in SN morphology using MRI may emerge as a promising diagnostic biomarker for PD. The SN is subdivided into pars compacta and pars reticulata, where the former contains a high density of neuromelanin (NM) containing dopaminergic cells. Using immunostaining for calbindin D_28K_, Damier et al. delineated calbindin-negative pockets/zones within the SNpc called 'nigrosomes' [26]. The greatest loss (~98%) of NM containing dopaminergic neurons was identified within a zone located in the caudal and medio-lateral SN labelled as 'nigrosome-1'– the largest of the five nigrosomes [27]. Accordingly, histological findings of healthy nigrosome-1 found high tyrosine hydroxylase, high NM, and low calbindin contents [28], while post-mortem imaging at 7T identified healthy nigrosome-1 as a hyperintense structure both on T2* and NM-sensitive T1 MRIs due to its low iron and high NM contents, respectively [28, 29]. In PD, however, this feature on 7T (and 3T) MRIs was virtually absent or significantly reduced possibly due to decreased NM or increased iron content, potentially providing a simple and specific diagnostic biomarker for PD [28–30]. Nigrosome-1 in PD was histologically found to be low in tyrosine hydroxylase and NM, consistent with the loss of melanized neurons in SNpc [28, 29]. Using automated volumetry on NM-sensitive 3T MRI, Castellanos et al. showed atrophy in the contralateral SNpc to have the highest sensitivity (91%) and specificity (89%) for differentiating PD from controls [30]. Similarly, using NM-sensitive 3T MRI, reduced area and width of T1 high signal in SN distinguished early-stage PD from essential tremor patients (sensitivity 66.7%, specificity 93.3%) [31]. Translating the findings to SW imaging, the healthy nigrosome-1 and surrounding neuroanatomy at the dorsolateral SN was found to visually resemble the tail of a swallow bird (the ‘swallow-tail’ sign) at 3T. This feature was lost in PD subjects and radiological assessments yielded a high diagnostic accuracy for PD compared to controls [32].

#### Structural MRI profiles of Lewy body spectrum disorders

Visible changes on conventional MRI are frequently non-specific and variable in DLB and PDD. Using VBM, a diffuse pattern of cortical atrophy involving temporal, occipital, right frontal and left parietal was identified in PDD versus normal controls [12]. When DLB and PDD groups were contrasted using VBM, Beyer et al. observed more prominent cortical reductions in temporal, occipital and parietal lobes in DLB patients [33]. Conversely, Burton et al. detected no such volumetric differences suggesting similar patterns of atrophy in the two closely-related α-synucleinopathies [12]. These variable findings may in part be attributable to pathological heterogeneity commonly observed in these disorders, such as the presence of concomitant AD pathology in DLB [5]. Indeed, more severe α-synuclein pathology and plaque burden were associated with progressively shorter duration of parkinsonism prior to dementia manifestation in PDD [34].

In PDD versus PD, more pronounced GM atrophy was identified in the occipital lobe and entorhinal cortex [12, 35]. Although hippocampal volumes alone may not differentiate between PD and PDD patients [35], starting from larger to smaller volumes they were found to be affected in a characteristic order: controls > PD > PD-MCI/PDD > AD [36] – a pattern that aligns with neuropathological evidence. The relative preservation of hippocampus (versus AD) is a supportive feature of DLB pathology, which has been incorporated into the DLB diagnostic criteria [4]. Notably, smaller hippocampal volumes may also be evident in DLB and even PD-MCI/PDD patients with concomitant AD pathology. Other investigations find reduced caudate and putaminal volumes in DLB and PD patients compared to AD and normal controls, although these differences were reported inconsistently [14, 37]. White matter (WM) hyperintensities may also be more frequent in PDD and DLB disorders (versus PD and controls), especially with coexisting AD pathology [38].

PD-MCI patients show reduced thalamic, amygdala and nucleus accumbens volumes compared to PD without MCI [39, 40]. In a longitudinal study, greater rates of cortical thinning were identified in PD-MCI patients in the temporal, occipital, parietal and supplementary motor area (SMA), relative to cognitively-stable PD and controls [40]. Involvement of SMA has been suggested as a specific biomarker of cognitive dysfunction in PD [40, 41]; whereas, marked occipital atrophy may be associated with the development of hallucinations in PD-MCI patients [41]. Interestingly, Weintraub et al. reported a baseline AD-type pattern of atrophy predictive of long-term cognitive decline, supporting the involvement of hippocampus and parietotemporal cortex in cognitive impairment in PD [42].

#### Structural MRI profiles of multiple system atrophy

**Fig. 1:**
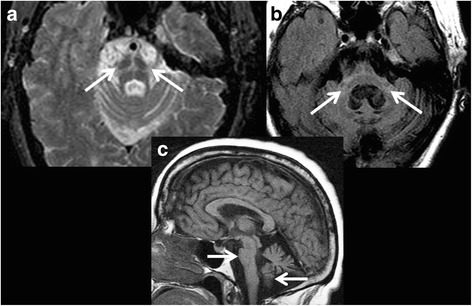
MRI of a patient with a clinical diagnosis of Multiple System Atrophy-*C*. **a** Axial proton density sequence at the level of the pons demonstrates cruciform pontine T2 hyperintensity consistent with the ‘hot cross bun’ sign secondary to selective vulnerability of the pontocerebellar tract in Multiple System Atrophy-*C*. Disproportionate atrophy of the pons and partially visualized cerebellar hemispheres is also evident. **b** Axial FLAIR sequence with cruciform T2 hyperintensity within the pons and middle cerebellar peduncles (i.e., ‘Middle Cerebellar Peduncle sign’) with marked atrophy. Cerebellar hemispheric and vermian atrophy is also seen with *ex vacuo* dilatation of the fourth ventricle. **c** Sagittal T1 sequence showing marked atrophy of the brainstem and cerebellar vermis

Several MRI-based features have been identified in MSA. In MSA-*P*, these characteristics on conventional MRI include: atrophy of the putamen, middle cerebellar peduncles (MCP), cerebellum, or pons; presence of a bilateral T2-hyperintense rim bordering the dorsolateral margins of the putamen (the ‘putaminal rim sign’); and T2-putaminal hypointensity. In MSA-*C*, atrophy of the putamen, MCP or pons may be evident, including the T2-hyperintensity of pons (the ‘hot-cross-bun sign’; Fig. 1a) [6]. T2-hyperintensity of MCP (the ‘MCP sign’; Fig. 1b) may also be observed in MSA [43]. Putaminal atrophy shows a high specificity (92.3%), but low sensitivity (44.4%) for distinguishing MSA-*P* from PD [44]. Meta-analysis of six studies (although heterogeneous) found putaminal volume to be significantly reduced in MSA patients versus PD, which may be helpful in the differential diagnosis [45]. Massey et al. found radiological assessment of MRI to be more accurate than the clinical diagnosis, and confirmed the ‘MCP sign’ and ‘hot-cross-bun sign’ as specific for MSA, albeit with a lower sensitivity [43]. Recently, a study compared the visual appearance of the ‘hot-cross-bun sign’ and showed it to be relatively clearer and of higher visual quality on T2* than more conventional T2-weighted images. T2* visual grade was comparable in possible and probable MSA-*C* patients suggesting improved utility to support the diagnosis at earlier stages [46]. Another study compared the T2 appearances of the ‘putaminal rim sign’ and T2-putaminal hypointensities on a 3T scanner and found these to be unhelpful in distinguishing MSA-*P*, PD and controls [44]. However, a combination of T2-putaminal hypointensity on gradient-echo sequence together with putaminal atrophy improved the diagnostic specificity of MSA-*P* to 98% (versus PD) and 95% (versus PSP), without altering the sensitivity [47]. Combined analysis of biomarkers may better differentiate MSA-*P* from PD and other atypical PS.

VBM and volumetric studies typically reveal striatonigral or olivopontocerebellar involvement in MSA patients. Schulz et al. identified volumetric loss in the striatum and brainstem regions in MSA versus PD and controls, although with considerable overlap with PSP patients [16]. Reduced cerebellar volume was a feature of both MSA-*P* and MSA-*C* subgroups [16]. Discriminant analysis allowed good separation of MSA from PD and controls in this study; however, MSA subtypes could not be reliably distinguished from PSP [16]. VBM in MSA-*P* patients showed GM loss in the left primary motor cortex (versus PD) and left SMA (versus controls) suggesting sensorimotor circuit involvement [48]. Similarly, Brenneis et al. detected cortical loss in the primary and supplementary motor areas as well as prefrontal and insular cortices bilaterally, with subcortical involvement of striatum and midbrain regions versus PD and controls [49]. In MSA patients with dementia, significant cortical thinning in the parahippocampal and lingual cortices was apparent versus non-demented MSA subjects [50]. Pontine atrophy is commonly observed in MSA [6, 43]; however, it is suggested that reduction in the area of pons over time may better discriminate MSA from PSP than cross-sectional volumetric assessment [51].

In a study applying a comprehensive quantitative MRI protocol (R1, R2 and R2* mapping, magnetization transfer and diffusion tensor imaging [DTI] techniques), the bilateral R2* increase in putamen best separated MSA-*P* patients from PD [52], consistent with SW imaging results demonstrating higher iron deposition in putamen versus PD [53].

#### Structural MRI profiles of progressive supranuclear palsy

**Fig. 2:**
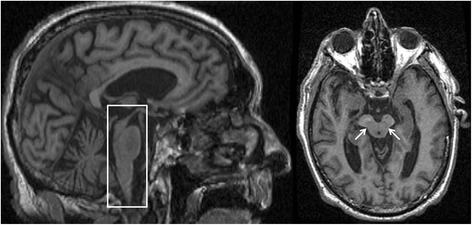
MRI of a patient with a clinical diagnosis of Progressive Supranuclear Palsy. The image on the left is a sagittal T1 sequence showing the ‘Hummingbird’ sign (box), while the axial T1 sequence on the right shows the ‘Morning Glory’ sign (arrows) both features seen in Progressive Supranuclear Palsy

Atrophy of the midbrain tegmentum and superior cerebellar peduncles (SCP) are frequently detected in PSP patients, compared to PD, MSA-*P*, CBS and controls [54, 55]. Numerous MRI-based features suggestive of PSP have been identified, including a midbrain diameter less than 17-mm, third-ventricle dilation, midbrain T2-hyperintensity, midbrain atrophy relative to pons (the ‘hummingbird sign’; Fig. 2) and the atrophy of midbrain tegmentum (the ‘morning glory sign’; Fig. 2). The hummingbird and morning glory signs were highly specific, but less sensitive than the clinical PSP diagnosis [43].

Compared with normal controls, PSP patients showed volumetric reductions in the mean whole-brain, brainstem, midbrain, and frontal GM [56]. All measured anatomical structures showed extensive overlap within the normal range, although overlap in midbrain volumes was considerably less on an individual basis [56]. VBM analysis in PSP identified neurodegenerative changes primarily in the midbrain, pons, thalamus and striatum versus controls, with midbrain structures more atrophic than those seen in CBS [54]. Other VBM studies of PSP revealed widespread cortical reductions in the prefrontal, frontal, insular, premotor and SMAs, as well as in the hippocampus and parahippocampal gyrus relative to controls. WM atrophy in PSP was detected in the pulvinar, thalamic, collicular, mesencephalic and frontotemporal regions [57, 58]. Relative to PD and controls, significant tissue loss in PSP was detected in the cerebral peduncles and midbrain, with minimal involvement of frontal regions (sensitivity 83%, specificity 79%) [59]. Frontal and midbrain atrophy rates, however, were associated with increasing executive and motor dysfunction in PSP, respectively [60]. In a pathology-proven sample, Joseph et al. revealed distinct patterns of atrophy in PSP compared to CBD patients [61]. Midbrain and SCP atrophy strongly suggested PSP, while frontoparietal and pallidum degeneration (without prominent midbrain atrophy) was indicative of CBD [61]. Interestingly, PSP patients with prominent extrapyramidal symptoms may show more midbrain atrophy. Conversely, less midbrain and more cortical/subcortical atrophy was found in PSP patients with dementia [61].

Ratios of the pons-to-midbrain area (P/M) and MCP-to-SCP widths (M/S) were significantly larger in PSP patients, relative to PD, MSA-*P* and controls [62]. Remarkably, the ‘magnetic resonance (MR) parkinsonism index’ derived from P/M and M/S ratios, proved 100% sensitive and specific for distinguishing PSP from PD, MSA-*P* and healthy controls [62]. Recently, the utility of midbrain-to-pons ratio was replicated in an autopsy-proven sample, wherein all non-PSP patients showed a value of greater-than 0.5 and the majority of PSP patients had a value less-than 0.5 [63]. Whether this measure is helpful in distinguishing patients at very early clinically undifferentiated stages, or in patients with PSP variants (e.g. PSP-*P*) where midbrain involvement may be less pronounced, is unknown. The midbrain atrophy rate predicts clinical decline over as short a time interval as 6 months, which may have potential as an effective outcome measure in PSP clinical trials [64].

#### Structural MRI profiles of corticobasal degeneration

**Fig. 3:**
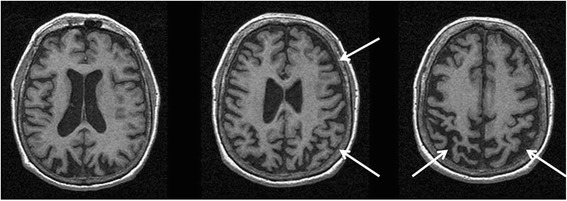
MRI of a patient with a pathological diagnosis of Corticobasal Degeneration. Serial axial T1 sequences showing right greater than left parietofrontal atrophy typical of that seen in Corticobasal Syndrome. In this case, the patient had a confirmed pathological diagnosis of Corticobasal Degeneration

Asymmetric atrophy of the frontoparietal cortices is typically observed in CBS/CBD patients contralateral to the more affected side of the body, although laterality may not exist in all cases (parietal > frontal atrophy in pathologically proven CBD case; Fig. 3). Using VBM, an asymmetric pattern of atrophy affecting the bilateral premotor cortex, superior parietal lobules, and striatum was detected in CBS versus controls [54]. Dorsofrontal and parietal cortical atrophy was found to be more pronounced in CBS versus PSP [54]. Similarly, Gröschel et al. reported parietal GM and WM to be significantly reduced in CBS compared to PSP and controls [56]. Another VBM study in early CBS subjects detected GM loss in the inferior frontal and premotor cortices, parietal operculum, superior temporal gyrus, and the hippocampus versus controls [65]. Parietal atrophy correlated with limb apraxia in this study [65]. Signal hyperintensities in the frontoparietal subcortical WM on T2/FLAIR images may also be present, although these changes are not consistently reported or specific to CBS. Notably, all of the above studies were conducted in samples that lacked pathological confirmation. Due to low diagnostic sensitivity in CBS [9], research in autopsy-proven samples may produce more precise results and are discussed below.

In an autopsy-proven sample, the degree of global atrophy on gross pathology was visibly more severe in CBD versus PSP [66] – a finding in line with MRI studies. Furthermore, the application of a pathological FTD staging scheme noted that the pattern of tissue loss in CBD was similar to other FTD-related tauopathies, while greater posterior corpus callosum degeneration was evident in CBD cases relative to FTD [66]. In another autopsy-proven sample of CBD patients clinically-diagnosed with CBS, Joseph et al. concluded that cortical atrophy, corpus callosum atrophy or periventricular WM changes on MRI do not demonstrate specificity for CBD [67]. Similarly, Whitwell et al. showed that the pattern of GM loss in CBS varies based on the true underlying pathology. In clinically-diagnosed CBS patients with pathologic diagnoses of FTD and AD, the GM loss was predominantly observed in the prefrontal cortex and parietal lobe, respectively [11]. Furthermore, focal loss in the premotor cortex and SMA was seen in both CBD and PSP patients, although more severe changes in these regions pointed towards CBD than PSP [11]. Using VBM in an autopsy-proven CBD sample, Lee et al. confirmed GM loss in the bilateral frontal cortex (including SMA), dorsolateral prefrontal cortex, pre- and post-central gyri, striatum, and brainstem, as compared to controls [68]. CBD can also present as a predominant extrapyramidal or cognitive syndrome. Cortical loss predominantly in the frontal lobes and insula with scarce WM atrophy was found in CBD with early dementia, whereas only moderate loss in these regions involving both GM and WM was evident in CBD with early extrapyramidal manifestations [61]. Thus, not unexpectedly, variable patterns of atrophy in CBD appear to correspond to the predominant clinical syndrome rather than the underlying cellular pathology.

### Diffusion tensor magnetic resonance imaging in Parkinsonian disorders

Diffusion tensor imaging is an *in vivo* tractography technique that allows indirect quantification of brain microstructural integrity by analyzing the overall displacement of water molecules (mean diffusivity [*D̄*]) and the degree of displacement in space (fractional anisotropy [FA]). In brain, water tends to move preferentially along the underlying microstructural WM tracts and this physical property can be used to measure *D̄* and FA, using voxel-based or region-of-interest approaches. Degeneration of WM tracts increases *D̄* while FA decreases, as the direction-dependent movement of water along the damaged tracts becomes restricted.

Decreased FA in the SN is commonly observed in PD patients, although its association with disease severity is unclear [69]. Using high-resolution DTI, greater FA reductions in caudal (than in middle or rostral) regions of the SN were identified, distinguishing PD from controls with 100% sensitivity and specificity [70]. Increased *D̄* in olfactory tracts and decreased FA in anterior olfactory structures have also been reported [71, 72], which are in line with olfactory disturbances seen in PD patients. Some studies find no measurable differences in *D̄* or FA in early PD versus normal subjects, possibly due to milder degenerative changes at early stages [73]. Atypical PS, as a group, may be distinguishable from PD by measuring increased *D̄* primarily in the corpus callosum, putamen, midbrain, as well as superior cerebellum and cerebellar peduncles [74].

In MSA-*P*, an elevated putaminal *D̄* was identified relative to PD, MSA-*C* and healthy controls [74, 75]. Remarkably, a combination of increased T2* relaxation rates and *D̄* in the putamen enabled discrimination of PD from MSA-*P* patients with 96% accuracy [75]. Likewise, Ito et al. found lower FA and increased apparent diffusion coefficient (ADC) values in MSA-*P* patients in putamen, cerebellum and pons, versus PD and controls [76]. FA and ADC in the pons proved to be highly specific (100%) for differentiating MSA-*P* patients from PD, and it was concluded that a combined analysis of pons, putamen and cerebellum might be more discriminatory than single-region analysis [76]. FA values in the corpus callosum and SCP did not differ between MSA and controls [77], whereas FA was markedly reduced in the MCP region versus PSP and controls [77, 78], correlating inversely with ataxia severity in MSA patients [77]. Cerebellar ataxia also correlated with elevated *D̄* values in the MCP and pons [78]. Marked FA decline was noted in the MCP, inferior cerebellar peduncle, and ventral pons among the MSA-*C* subjects versus controls [79]. Pellecchia et al. compared tractographic changes in MSA variants and reported increased ADC values in the putamen and pons in MSA-*P* (versus MSA-*C* and controls), and in the cerebellum and MCP in MSA-*C* subjects (versus MSA-*P* and controls), which may be helpful as biomarkers of microstructural injury in these disorders [80].

Compared with healthy subjects, DTI studies in PSP patients may show: increased *D̄* or ADC in the decussation of SCP, thalamus, cingulum, motor and SMA [78, 81]; decreased FA and increased ADC in the frontal part of inferior frontooccipital fasciculus [82]; and decreased FA in the orbitofrontal WM, anterior cingulum and motor area [81], as well as in the superior longitudinal fasciculus, arcuate fasciculus, posterior thalamic radiations and internal capsule [58]. Elevated *D̄* in the midbrain and SCP distinguished PSP from other atypical PS [74]. Blain et al. reported increased *D̄* in the decussation of SCP in PSP compared to MSA, PD and controls [78]. Seppi et al. also found raised ADC values in the putamen, globus pallidus and caudate nucleus, which discriminated PSP from PD (sensitivity 90%, specificity 100%) [83]. Using serial MRIs two years apart from each other, a significant increase in putaminal ADC was detected in PSP versus controls (with no measurable change in MSA patients), suggesting putaminal changes over time as a potential differentiating biomarker [51].

In CBS relative to controls, decreased FA was identified in the long frontoparietal connecting tracts, intraparietal associative fibers, corpus callosum and sensorimotor cortical projections [65]. FA abnormalities in frontoparietal associative fibers correlated with limb apraxia, while the limb-kinetic measure of apraxia correlated with FA values in the hand sensorimotor connecting fibers [65]. Corpus callosum DTI abnormalities may be useful in differentiating CBS from PD, as increased *D̄* and decreased FA were observed in the posterior truncus of corpus callosum, reflecting neurodegenerative changes in transcallosal connectivity [73].

Relative to controls, DTI studies in DLB show abnormalities in the corpus callosum, dorsal striatum, frontal, parietal and occipital WM tracts [84], as well as in amygdala and inferior longitudinal fasciculus with less temporal involvement [85]. Conversely, AD patients show reduced FA and elevated *D̄* in the medial temporal lobe structures (especially the hippocampus). Decreased FA was detected in the precuneus in DLB versus AD [86], and in the posterior cingulate bundles in PDD versus PD [87]. Elevated *D̄* in the longitudinal fasciculus was exclusively found in DLB patients with hallucinations relative to DLB without hallucinations [85].

### Proton magnetic resonance spectroscopy

Proton magnetic resonance spectroscopy (^1^H-MRS) is a non-invasive *in vivo* imaging technique that relies upon the resonance frequencies of protons to estimate the amount of biochemical molecules in brain. The relative concentrations of proton-containing metabolites appear as peaks on a neurospectrograph near their characteristic resonance frequencies. Using MRS, the following metabolites are commonly assessed in neurodegenerative disorders: (a) N-acetyl aspartate (NAA) – an indicator of neuronal health, integrity and metabolism; (b) Choline-containing compounds, primarily free choline, phosphorylcholine and glycerophosphorylcholine – markers of membrane turnover, osmoregulation and inflammation; (c) myo-inositol – a signature of gliosis, demyelination and osmoregulation, and (d) Creatine (Cr), mix of creatine and phosphocreatine – a reference standard with relatively stable levels in healthy brain and is used to normalize the spectral data for comparative purposes [88].

In PD, reduced NAA or NAA/Cr levels were observed in the lentiform nucleus (LN) (putamen and globus pallidus), temporoparietal and posterior cingulate cortex as well as in the pre-SMA relative to normal controls [88–91]. There have been inconsistent reports of a correlation between NAA/Cr ratios in these regions with disease severity or duration [88–91]. Using 3D MRS, Gröger et al. noted higher NAA/Cr ratio in the rostral (versus caudal) SN in atypical PS and controls, whereas this pattern was reversed in PD suggesting pathological neuronal loss in the rostral SNpc [92]. PSP patients showed reductions in NAA/Cr ratios in the LN, brainstem, centrum semiovale, frontal and pre-central regions relative to controls [93, 94], although more severe reductions were noted in the putamen (versus PD and MSA) [86] and frontal cortex (versus PD) [88, 95]. Lower NAA/Cr ratios were also noted in the putamen and pontine base in MSA patients compared to PD and controls [96]. CBS subjects showed reductions in NAA/Choline or NAA/Cr levels in the frontoparietal cortex, LN and centrum semiovale contralateral to the more affected side [94, 97]. NAA/Cr reductions in CBS were pronounced in the frontal cortex and putamen relative to PD, MSA and vascular parkinsonism with clear putaminal asymmetry [95]. Notably, lower NAA/Cr values in the putamen are found in PSP subjects as well, and laterality observed in CBS may be helpful in differentiating the two disorders (although, this may simply affirm what is apparent on clinical examination). In fact, putaminal NAA levels were found to be reduced across PD, MSA, PSP and CBS patients versus controls, although to varying degrees within each of these parkinsonian syndromes. Patients with DLB and PDD may exhibit lower NAA/Cr values in the posterior cingulate gyrus and medial temporal lobe structures, but to a lesser degree than in AD [98, 99].

In a recent study, Mazuel et al. showed restoration of total NAA and Cr levels in putamen with no change in total myo-inositol levels in PD patients undergoing L-DOPA treatment. The total NAA, Cr and myo-inositol levels were lower in these patients in the off-drug condition versus normal controls [100]. Another study found an association between parkinsonism severity (via Hoehn and Yahr staging) and putaminal NAA/Cr ratios in atypical PS patients (Table 1) [95].

**Table 1:** Common magnetic resonance imaging findings in Parkinson’s disease and atypical parkinsonian syndromes

Neuropathology	Disorders	MRI signs	Structural/volumetric MRI findings	Diffusion Tensor MRI findings	Proton MRS findings
Synucleinopathies	PD	1. Swallow tail sign 2. Loss of nigrosome-1	↓ in frontal lobe, hippocampus, anterior cingulate and superior temporal gyri, and olfactory bulb and tract volumes vs. HC [12, 13, 17] ↓ in orbitofrontal, ventrolateral, prefrontal and occipitoparietal cortex vs. HC [15] ○ or ↓ in caudate, putamen and brainstem volumes vs. HC [14–16] ↑ R2* transverse relaxation rate in SN vs. HC [19, 25]	DTI may be normal in early-PD vs. HC [73]. ↓ FA in SN and anterior olfactory structures; and ↑ *D̄* in olfactory bulb and tracts vs. HC [69–72]. ↑ in *D̄* primarily in corpus callosum, putamen, midbrain, cerebellum and cerebellar peduncles may distinguish atypical PS from PD [74].	↓ in NAA and NAA/Cr levels in LN, temporoparietal and posterior cingulate cortex, and in pre-SMA vs. HC [88–91]
	PDD/DLB (LBD)		↓ in temporal, occipital, frontal and parietal cortices in PDD vs. HC [12, 27]. ↓ in temporal, occipital and parietal cortices may be seen in DLB vs. PDD [33] ↓ in occipital and entorhinal cortices in PDD vs. PD [12, 35]. ↓ in thalamic, amygdala and nucleus accumbens volumes, and ↑ in rate of temporal, occipital, parietal and SMA cortical thinning in PD-MCI vs. PD-non-MCI [39, 40]	↑ WM abnormalities in corpus callosum, dorsal striatum, frontal, parietal and occipital regions, as well as in amygdala and inferior longitudinal fasciculus in DLB with less temporal involvement vs. HC [84, 85]. ↓ FA in parietal lobe (precuneus) in DLB vs. AD [86].	↓ in NAA/Cr values in the posterior cingulate gyrus and medial temporal lobe structures in DLB and PDD, although to a lesser degree than in AD [98, 99].
	MSA	1. Putaminal rim sign 2. Hot-cross-bun sign 3. MCP sign	↓ in putamen, MCP, cerebellum, pons and striatal volumes in MSA-*P* and MSA-*C* vs. HC [6, 16]. ↓ in putaminal, cerebellar and pontine volumes in MSA vs. PD [16, 44, 45] ↓ in primary and SMA, prefrontal and insular cortices, striatum and midbrain in MSA-*P* vs. PD and HC [49] ↑ cortical thinning in parahippocampal and lingual cortex in MSA-demented vs. MSA-non-demented [50]	↑ putaminal *D̄* in MSA-*P* vs. PD, MSA-*C* and HC [74, 75]. ↓ FA and ↑ ADC in MSA-*P* in putamen, cerebellum and pons vs. PD and HC [76]. ↓ FA in MCP, inferior cerebellar peduncle, and ventral pons in MSA-*C* vs. HC [79]. ↓ FA and ↑*D̄* in MCP and pons vs. HC [77, 80]. ↑ ADC in cerebellum and MCP in MSA-*C* vs. MSA-*P* and HC [80]; ↓ FA in MCP vs. PSP and HC [77, 78]	↓ in NAA/Cr ratio in putamen and pontine base in MSA vs. PD and HC [96].
Tauopathies	PSP	1. Hummingbird sign 2. Morning glory sign	↓ in prefrontal, frontal, insular, premotor, SMA, hippocampus and parahippocampal regions; ↓ WM in pulvinar, thalamus, colliculus, mesencephalon and frontotemporal regions; ↓ in midbrain, pons, thalamus and striatum, vs. HC [57, 58]. ↓ in midbrain and SCP volumes vs. PD, MSA-*P*, CBS and HC [54, 55, 59, 61, 62] ↓ in brainstem, midbrain and frontal cortex vs. HC [56] ↓ midbrain atrophy and ↑ cortical/subcortical atrophy in PSP with dementia [61]	↑ *D̄* or ADC in decussation of SCP, thalamus, cingulum, motor and SMA; ↓ FA in the frontal inferior frontooccipital fasciculus, superior longitudinal fasciculus, arcuate fasciculus, posterior thalamic radiations, internal capsule, orbitofrontal WM, anterior cingulum, motor area vs. HC [58, 78, 81, 82] ↑ ADC in putamen and pons in MSA-*P* vs. MSA-*C* and HC [80] ↑ *D̄* in midbrain and SCP vs. atypical PS group [74]. ↑ ADC in putamen, globus pallidus and caudate nucleus vs. PD [83].	↓ in NAA/Cr ratio in LN, brainstem, centrum semiovale, frontal and precentral cortex vs. HC [93, 94]. Relatively greater ↓ in NAA/Cr ratio in putamen and frontal cortex vs. PD [86, 88, 95].
	CBD/CBS		↑ global brain atrophy in CBD vs. PSP [66] ↓ in bilateral frontal cortex (including SMA), dorsolateral prefrontal cortex, pre/post-central gyri, striatum, and brainstem in CBD vs. HC [68] ↓ in frontal and insula cortex with scarce WM atrophy in CBD-dementia; moderate ↓ in GM/WM of these regions in CBD with early extrapyramidal manifestations [61] ↓ in prefrontal cortex and parietal lobe, respectively, in CBD-FTD and CBD-AD [11]	↓ FA in the long frontoparietal connecting tracts, intraparietal associative fibers, corpus callosum and sensorimotor projections of cortical hand areas, in CBS vs. HC [65]. ↓ FA and ↑ *D* in posterior truncus of corpus callosum may differentiate CBS from PD [73].	↓ in NAA and NAA/Cr levels contralaterally in frontoparietal cortex, LN, centrum semiovale and putamen, in CBS vs. HC [94, 95, 97]. Greater ↓ in NAA and NAA/Cr levels in frontal cortex and putamen with marked putaminal asymmetry, in CBS vs. PD, MSA and vascular parkinsonism [95]

Legend: ○ normal; ↓ decrease; ↑ increase

Abbreviations: *AD*, Alzheimer’s disease; *ADC*, apparent diffusion coefficient; *CBD*, pathologically-proven corticobasal degeneration; *CBS*, clinically-diagnosed corticobasal syndrome; *D̄*, mean diffusivity; *DLB*, dementia with Lewy bodies; *DTI*, diffusion-tensor imaging; *FA*, fractional anisotropy; *FTD*, frontotemporal degeneration; *HC*, healthy controls; *LBD*, Lewy body spectrum disorders; *LN*, lentiform nucleus; *MCI*, mild cognitive impairment; *MCP*, middle cerebellar peduncle; *MRS*, magnetic resonance spectroscopy; *MSA*, multiple system atrophy; *MSA-P*, MSA-parkinsonian type; *MSA-C*, MSA-cerebellar type; *NAA*, N-acetyl aspartate; *NAA/Cr*, NAA-to-creatine ratio; *PD*, Parkinson’s disease; *PDD*, Parkinson’s disease dementia; *PS*, parkinsonian syndromes; *PSP*, progressive supranuclear palsy; *SCP*, superior cerebellar peduncle; *SMA*, supplementary motor area; *SN*, substantia nigra; *WM*, white matter

## Single photon emission computed tomography in Parkinsonian disorders

SPECT is a functional nuclear imaging technique predominantly used in PS to assess the integrity of nigrostriatal dopaminergic system as well as to detect cerebral perfusion changes *in vivo*. SPECT employs selective gamma-emitting cocaine analogues, such as ^123^I-*N*-ω-fluoropropyl-2β-carbomethoxy-3β-(4-iodophenyl) nortropane (^123^I-FP-CIT or ^123^I-ioflupane), which bind to striatal dopamine transporters (DAT), as well as ligands for dopamine D2 receptors, such as ^123^I-(S)-(−)-2-hydroxy-3-iodo-6-methoxy-*N*-([1-ethyl-2-pyrrolidyl]-methyl) benzamide) (^123^I-IBZM). DAT is a sodium-coupled transmembrane protein that mediates the reuptake of dopamine from the synaptic cleft, and is localized to presynaptic nigrostriatal terminals. Striatal dopamine D2 receptors are G-protein-coupled inhibitory receptors expressed both at the dopamine target cells postsynaptically, as well as presynaptically on the nigrostriatal axonal terminals [101]. SPECT-based imaging of dopamine D2 receptors evaluates the post-synaptic functioning of striatal neurons. ^123^I-FP-CIT is the most widely utilized ligand to measure DAT density due to its faster kinetics, greater selectivity and compatibility with levodopa treatment. Cerebral perfusion changes can be measured via SPECT using lipophilic radiotracers, such as ^99m^Tc-ethyl-cysteinate-diethyl-ester (^99m^Tc-ECD) and ^99m^Tc-hexamethyl-propylene-amine-oxime (^99m^Tc-HMPAO), which can traverse the blood-brain barrier, perfuse brain tissues, and therefore their uptake is proportional to cerebral blood flow (Table 2).

**Table 2:** Common radionuclides used in SPECT, PET and myocardial scintigraphy imaging

Radionuclide	IUPAC name	Main Target or Measure
**SPECT**		
*Nigrostriatal Integrity*		
^123^I-FP-CIT (^123^I-ioflupane)	^123^I-*N*-ω-fluoropropyl-2β-carbomethoxy-3β-(4-iodophenyl)nortropane	Presynaptic DAT
^123^I-β-CIT	^123^I-2β-carbomethoxy-3β-(4-iodophenyl)tropane	Presynaptic DAT
^123^I-IPT	^123^I-*N*-(3-iodopropen-2-yl)-2β-carbomethoxy-3β-(4-chlorophenyl)tropane	Presynaptic DAT
^99m^Tc-TRODAT-1	^99m^Tc-[2-[[2-[[[3-(4-chlorophenyl)-8-methyl-8-azabicyclo [1–3] oct-2-yl]methyl](2-mercaptoethyl)amino]ethyl]amino]ethanethiolato(3-)-N2,N2′,S2,S2′]oxo-[1R-(exo-exo)]	Presynaptic DAT
^123^I-IBZM	^123^I-(S)-(−)-2-hydroxy-3-iodo-6-methoxy-*N*-([1-ethyl-2-pyrrolidyl]-methyl)benzamide)	Postsynaptic D2 receptors
^123^I-IBF	^123^I-(S)-5-iodo-7-*N*-[(1-ethyl-2-pyrrolidinyl)methyl]carboxamido-2,3-dihydrobenzofuran	Postsynaptic D2 receptors
^123^I-epidepride	^123^I-(S)-*N*-((1-ethyl-2-pyrrolidinyl)methyl)-5-iodo-2,3-dimethoxybenzamide	Postsynaptic D2 receptors
*Cerebral Perfusion*		
^99m^Tc-ECD	^99m^Tc-ethyl cysteinate diethylester	Cerebral perfusion
^99m^Tc-HMPAO	^99m^Tc-hexamethylpropyleneamineoxime	Cerebral perfusion
^123^I-IMP	^123^I-*N*-isopropyl-*p*-iodoamphetamine	Cerebral perfusion
**PET**		
*Nigrostriatal Integrity*		
^18^F-dopa	3,4-dihydroxy-6-^18^F-fluoro-L-phenylalanine	Presynaptic DAT
^18^F-FE-PE2I	^18^F-(E)-*N*-(3-iodoprop-2-enyl)-2β-carbofluoroethoxy-3β-(4′-methyl-phenyl)nortropane	Presynaptic DAT
^11^C/^18^F-β-CFT	^11^C/^18^F-2-β-carbomethoxy-3-β-(4-fluorophenyl)tropane	Presynaptic DAT
^11^C-methylphenidate	^11^C-methylphenidate	Presynaptic DAT
^11^C/^18^F-DTBZ	^11^C/^18^F-dihydrotetrabenazine	Presynaptic VMAT2
^18^F-FP-(+)-DTBZ	^18^F-(+)-fluoropropyldihydrotetrabenazine	Presynaptic VMAT2
^11^C-raclopride	^11^C-3,5-dichloro-*N*-[[(2S)-1-ethylpyrrolidin-2-yl]methyl]-2-hydroxy-6-methoxybenzamide	Postsynaptic D2 receptors
*Cerebral Amyloid*		
^11^C-PIB	2-(4-*N*-^11^C-methylaminophenyl)-6-hydroxybenzothiazole	Cerebral amyloid
^18^F-florbetaben	4-{(*E*)-2-[4-(2-{2-[2-^18^F-Fluoroethoxy]ethoxy}ethoxy)phenyl]vinyl}-*N*-methylaniline	Cerebral amyloid
*Cerebral Tau*		
^18^F-T807 (^18^F-AV-1451)	7-(6-fluoropyridin-3-yl)-5H-pyrido[4,3-b]indole	Cerebral tau
^18^F-FDDNP	2-(1-(6-[(2-^18^F-fluoroethyl)(methyl)amino]-2-naphthyl)ethylidene)malononitrile	Cerebral tau
^18^F-THK523	2-(4-aminophenyl)-6-(2-^18^F-fluoroethoxy)quinoline	Cerebral tau
^18^F-THK5105	6-[(3-^18^F-fluoro-2-hydroxy)propoxy]-2-(4-dimethyl-aminophenyl)quinolone	Cerebral tau
^11^C-PBB3	^11^C-labelled phenyl/pyridinyl-butadienyl-benzothiazoles/benzothiazolium derivative	Cerebral tau
*Cerebral Metabolism*		
^18^F-FDG	^18^F-fluoro-2-deoxyglucose	Glucose metabolism
*Neuroinflammation*		
^11^C-(*R*)-PK11195	^11^C-1-(2-chlorophenyl-N-methylpropyl)-3-isoquinolinecarboxamide	Mitochondrial TSPO
^11^C-DPA-713	^11^C-*N*,*N*-diethyl-2-[2-(4-methoxyphenyl)-5,7-dimethyl-pyrazolo[1,5-a]pyrimidin-3-yl]-acetamide	Mitochondrial TSPO
**Myocardial Scintigraphy**		
*Dysautonomia*		
^123^I-MIBG	^123^I-metaiodobenzylguanidine	Cardiovascular dysautonomia

### Presynaptic dopamine transporter imaging

Imaging presynaptic DAT with SPECT (DAT-SPECT) has been investigated for its utility in the differential diagnosis of PS. Normal DAT binding using ^123^I-FP-CIT appears as two intense symmetric ‘comma-shaped’ regions of activity in the striatum (caudate anteriorly and putamen posteriorly); whereas, an abnormal scan may fall into one of four types: (a) asymmetrically reduced putaminal activity, (b) symmetrically reduced putaminal activity with relative preservation of caudate activity, (c) virtual absence of putaminal activity associated with reduced caudate activity unilaterally or bilaterally, and (d) fairly uniform involvement of putamen and caudate unilaterally or bilaterally [102, 103]. SPECT shows normal density of presynaptic DAT in healthy controls, patients with essential tremor and in drug-induced or psychogenic parkinsonism [104–106], whereas reduced DAT uptake is indicative of nigrostriatal degeneration and is detected in PD, PDD, MSA and PSP patients versus controls [105, 107, 108]. DAT binding was also significantly impaired in DLB and PD patients versus AD and controls [109, 110]. Loss of DAT is typically more pronounced in the hemisphere contralateral to the parkinsonian symptoms/signs and tends to appear symmetric in patients with symmetric motor deficits [111]. The posterior putamen shows earlier and more severe signal loss than the anterior putamen or caudate in PD [111]. Significant correlations of striatal DAT-SPECT binding with the Hoehn & Yahr disease stage, UPDRS motor score, and with the UPDRS bradykinesia subscale have been demonstrated, although no associations with rigidity or tremor were observed [104, 112–114].

A meta-analysis confirmed the utility of DAT-SPECT for the differential diagnosis of early PD from healthy controls, patients with essential tremor, and vascular parkinsonism with high accuracy [115]. A multi-centered study evaluating the utility of visual assessment of ^123^I-FP-CIT SPECT reported a sensitivity of 97% for clinically-diagnosing parkinsonism and a specificity of 100% for reliably excluding essential tremor cases across institutions [116]. In a longitudinal study, Nocker et al. reported higher rates of signal reductions in the caudate and anterior putamen in MSA-*P* patients relative to PD – a finding consistent with faster rate of disease progression in MSA-*P* [117].

Several investigations suggest a different pattern of DAT loss in PSP. In contrast to PD and MSA-*P*, a more symmetric pattern of DAT loss was observed [104, 118, 119], with an index of asymmetry significantly higher in PD than in PSP [119]. Lower striatal-to-occipital, but higher putamen-to-caudate binding ratios were found in PSP versus PD [118, 119], indicating a relatively uniform involvement of striatal dopamine neurons in PSP. Using ^123^I-*N*-(3-iodopropen-2-yl)-2β-carbomethoxy-3β-(4-chlorophenyl) tropane (^123^I-IPT), Im et al. confirmed that PSP patients exhibit more pronounced but fairly uniform DAT loss in the striatal regions-of-interest versus PD. In comparison, PD patients demonstrated lower DAT reductions (or higher signal) in the caudate head and caudate/putamen transitional region relative to putamen, with smaller posterior putamen-to-caudate binding ratios [107]. Despite these findings, the pattern of striatal DAT loss has not proven reliable in differentiating parkinsonian disorders on an individual case basis.

DAT-SPECT has a useful role in distinguishing DLB from other forms of dementia in uncertain cases. Abnormal DAT scan in patients clinically-diagnosed as ‘possible’ DLB suggests a revised diagnosis to ‘probable’ DLB at a 12-month follow-up [120]. Using ^123^I-FP-CIT, a longitudinal study with neuropathological confirmation reported 88% sensitivity and 100% specificity of diagnosing DLB versus AD – accuracy superior than clinical diagnosis alone (sensitivity 75%, specificity 42%) [121]. A meta-analysis evaluating both visual and semi-quantitative studies supported these findings showing sensitivity and specificity of ^123^I-FP-CIT to be greater than 80% in differentiating DLB from other dementia syndromes of AD and FTD. These values greatly improved when neuropathological diagnosis was used as a reference standard (sensitivity 87%, specificity 92%) [122]. These results suggest that the clinical diagnosis of DLB can be improved upon using ^123^I-FP-CIT SPECT. Another meta-analysis reported similar accuracies in differentiating DLB from a non-DLB group (sensitivity 86.5%, specificity 93.6%), although studies employing different analytic and diagnostic methodologies were pooled and assessed together in this analysis [123]. Finally, CBS patients showed DAT reductions in the striatum, but with greater hemispheric asymmetry than in PD [124, 125]. In a study of two pathologically-proven CBD cases, asymmetry of DAT loss was found to be significantly worse on follow-up scan and maybe of diagnostic value in possible CBD patients [125].

Interestingly, about 10-20% of PD patients, enrolled in neuroprotective trials of PD undergoing DAT imaging, were found to have ‘scans without evidence of dopaminergic deficit’ (acronym: SWEDD) [126]. Follow-up studies so far have established SWEDD as a relatively heterogeneous group, with the following main conclusions: 1) most cases represented a clinical misdiagnosis of PD (commonly dystonia), 2) some cases were false-negatives with true PD, as evidenced by abnormal follow-up scan and a positive levodopa response, 3) initial imaging reports may have been inaccurate in some due to practical/methodological issues, and 4) accurate diagnoses in many cases remains unclear due to lack of neuropathological confirmation [126–128].

### Postsynaptic dopamine D2 receptor imaging

Using SPECT with ^123^I-IBZM and ^123^I-(S)-5-iodo-7-*N*-[(1-ethyl-2-pyrrolidinyl) methyl] carboxamido-2,3-dihydrobenzofuran (^123^I-IBF) as ligands, binding potentials for postsynaptic D2 receptors were identified to be within the normal range in levodopa-treated PD as well as in patients with essential tremor and DLB [105, 111]. Conversely, reduced binding potentials were detected among MSA and PSP patients versus controls [105, 129]. Striatal D2 receptors were upregulated in drug-naïve PD patients, likely in response to nigrostriatal denervation with the greatest increase in the posterior putamen [111, 130]. Studies generally find the density of D2 receptors to be preserved among CBS patients, although this finding was not reliably shown on an individual case-to-case basis. Using ^123^I-IBZM as a tracer, Klaffke et al. [108], Pirker at al. [131] and Plotkin at al. [105] respectively reported 7 out of 8 (7/8), 8/9 and 7/9 clinically-diagnosed CBS patients with normal D2 bindings, suggesting preservation of dopamine D2 receptors. It is important to consider that a normal D2 SPECT scan may not exclusively confirm or discount an atypical PS. Further studies with pathologically-proven samples are warranted to determine the true sensitivity and specificity of D2 SPECT in distinguishing atypical PS.

Striatal region-of-interest analysis using D2 SPECT ligands revealed that the ratios of posterior putamen to caudate binding were > 1 in almost all drug-naïve PD cases, levodopa-treated PD and PSP patients. In contrast, this ratio was < 1 in 5/7 MSA patients, implicating a more pronounced loss of D2 receptors in the posterior putamen of MSA individuals [111]. Further research is necessary to clarify and better understand the role of D2 receptor binding in the differential diagnosis of PS.

Some studies examined the utility of combining presynaptic DAT imaging with postsynaptic D2 receptor SPECT in an effort to improve the diagnostic accuracy. A meta-analysis, however, reported the diagnostic accuracy of SPECT using both pre- and postsynaptic tracers to be relatively low [115]. Koch et al., on the other hand, demonstrated a gain of diagnostic power using a dual tracer model that integrated both striatal ^123^I-IBZM D2 receptor binding together with presynaptic DAT imaging via ^123^I-FP-CIT. This model discriminated PD from atypical PS with 90.3% sensitivity and 73.9% specificity, superior than using striatal D2 receptor binding alone [132].

### Cerebral perfusion studies

Using ^99m^Tc-HMPAO as a tracer in DLB, regional hypoperfusion was detected in the parietal, temporal and occipital regions relative to controls [133]. Upon comparison with AD, occipital hypoperfusion in DLB patients was the only differentiating feature in this study [133]. Occipital hypoperfusion in DLB patients has also been observed using other tracers, including *N*-isopropyl-p-^123^I-iodoamphetamine (^123^I-IMP), and ^99m^Tc-ECD. Hypoperfusion in PD using SPECT was found in the frontal lobe and occipital cortex versus controls [134, 135], whereas increased perfusion was detected in the primary sensorimotor cortex [136]. This frontal lobe hypoperfusion was also evident in a one-year follow-up study in PD [137]. Song et al. compared PD and MSA-*P* subjects using ^123^I-IMP tracer and reported frontal cortex hypoperfusion in both disorders, although occipital hypoperfusion was exclusive to PD [136]. Conversely, putaminal hypoperfusion was evident in MSA-*P* patients relative to PD [136]. Decreased perfusion in PDD (versus controls) was found in all cortical areas, particularly the temporal and parietal regions [135]. In MSA-*C* patients, hypoperfusion together with local atrophy was detected in the cerebellum and pons compared to controls [138].

## Positron emission tomography in Parkinsonian disorders

Positron emission tomography (PET) is another *in vivo* functional neuroimaging technique that utilizes a variety of radionuclides to elucidate the integrity of the dopaminergic system, cerebral metabolism, pathological protein accumulation, and inflammation in the brain. Radiotracers, such as ^18^F-dopa and ^11^C-raclopride, can be employed to image the integrity of presynaptic and postsynaptic nigrostriatal projections, respectively. The functioning of the pre-synaptic monoaminergic system can be evaluated using ^11^C-dihydrotetrabenazine (^11^C-DTBZ) or ^18^F-labelled analogues. Cerebral glucose metabolism is commonly assessed using ^18^F-labelled fluorodeoxyglucose (^18^F-FDG) tracer, where reduced uptake is suggestive of lower regional tissue metabolism. Amyloid burden in the brain has been widely assessed using an ^11^C-labelled thioflavin analogue, known as the Pittsburgh compound B (^11^C-PIB), as well as using other ^18^F-labelled ligands. Finally, tau imaging is a newer technique that is still in its infancy and is aimed at detecting abnormally-folded tau deposits in AD and other tauopathies.

### Presynaptic and postsynaptic dopaminergic imaging

Imaging the nigrostriatal dopaminergic system using PET provides helpful diagnostic information and complements SPECT findings. ^18^F-dopa is a well-known presynaptic PET tracer that measures the density of presynaptic nigrostriatal axons, specifically, the activity of the nigrostriatal aromatic amino acid decarboxylase (AADC) protein – an enzyme that converts ^18^F-dopa to ^18^F-dopamine, and provides an indirect estimation of the dopaminergic storage pool. In PD, a decrease in ^18^F-dopa uptake is first observed in the posterior putamen, followed by the anterior putamen and caudate nucleus, contralateral to the clinically affected side [139, 140]. Striatal ^18^F-dopa uptake was found to be associated with PD progression, while putaminal ^18^F-dopa uptake (but not caudate nucleus) showed an association with motor severity [140]. ^18^F-dopa activity was also reduced in putamen in atypical PS versus controls [141–143], but with a more severe decline in the caudate head relative to PD [142]. Similarly, Brooks et al. detected depressed striatal ^18^F-dopa uptake in PD, PSP and MSA versus controls [141]. In contrast to PD, however, putamen and caudate regions were equally impaired in PSP, and the mean uptakes in these regions among MSA patients were in between that of PD and PSP [141]. Similar to DAT-SPECT findings, striatal DAT PET studies have also found abnormal uptake in PD, MSA-*P*, PSP and DLB groups versus controls, whereas normal activity was detected in patients with essential tremor and MSA-*C* [144, 145]. Using dual-phase ^18^F-FP-CIT PET, Jin et al. found that visual interpretation of early-phase images (acquired at 5-min) to have a favourable diagnostic potential for distinguishing PD from atypical PS (sensitivity 75.4%, specificity 100%) [144]. Using a recently-developed PET radiotracer, ^18^F-(E)-N-(3-iodoprop-2-enyl)-2β-carbofluoroethoxy-3β-(4′-methyl-phenyl)nortropane (^18^F-FE-PE2I), reduction in DAT in the striatum and SN was replicated in PD patients versus controls. A shorter acquisition time (~22 min) and favorable kinetics were emphasized as advantages of ^18^F-FE-PE2I compared to traditional radionuclides [145]. Importantly, presynaptic tracers may not precisely estimate the nigrostriatal dopaminergic injury due to ongoing compensatory mechanisms, including the up-regulation of AADC activity and down-regulation of presynaptic DAT in response to neurodegeneration [146].

^11^C-DTBZ tracer or (its ^18^F-labelled analogues) labels the vesicular monoamine transporter type-2 (VMAT2), important for packaging and storing monoamines (e.g. dopamine) into synaptic vesicles. ^11^C-DTBZ PET have shown decreased striatal VMAT2 binding in PD reflecting nigrostriatal degeneration, accompanied by rather minimal compensatory changes [146]. Conversely, the binding potential for ^11^C-methylphenidate (DAT ligand) was reduced to a much greater extent relative to ^11^C-DTBZ, suggesting marked compensatory down-regulation of striatal DAT activity [146]. Using a novel ^18^F-tetrabenazine derivative [^18^F-FP-(+)-DTBZ or ^18^F-AV-133], Okamura et al. detected the greatest regional decrease in VMAT2 binding in the posterior putamen, followed by anterior putamen and caudate nucleus in PD [147].

^11^C-raclopride is a PET tracer that binds to striatal post-synaptic D2 receptors. In untreated PD patients, D2 binding potentials may appear normal or upregulated contralateral to the clinically affected side versus controls [148–150], whereas, reductions are more commonly seen in medicated PD [148], as well as in atypical PS patients [150]. Reduced ^11^C-raclopride binding differentiated all medicated PD patients from healthy controls [151]. Binding was found to be reduced in PSP patients (versus controls) [150], and MSA patients (versus PD and controls) [143, 151], which correlated with striatal glucose hypometabolism in MSA [151]. Van Laere et al. contrasted ^11^C-raclopride binding potentials in MSA-*P* and PD subjects, and identified the caudate-to-putamen and anterior-to-posterior putamen binding potential ratios to be significantly higher in MSA-*P* patients than in PD [152]. Consistent with striatal D2 SPECT studies [111], this result suggests bilateral D2 receptor loss in putamen of MSA-*P* patients, especially in the posterior part. Mean ^11^C-raclopride local influx ratios were also decreased in the bilateral pons, bilateral cerebellum, and posterior putamen in MSA-*P* patients versus PD (albeit with extensive overlap) – a finding consistent with brain volumetric, perfusion and metabolic studies in MSA. Discriminant analysis that combined ^11^C-raclopride striatal binding potentials with local influx ratios improved discrimination between MSA-*P* and PD patients with 100% accuracy (when normal controls were excluded from the analysis) [152].

### Glucose metabolism

In PD, ^18^F-FDG-PET often reveals relatively preserved glucose metabolism in the LN and thalamus [153, 154], and hypometabolism in the bilateral parietal, premotor and supplementary motor regions relative to controls [153, 155]. This preserved metabolism in the basal ganglia may distinguish PD patients from MSA and PSP, where a corresponding metabolic decline is commonly observed. MSA patients exhibit impaired glucose metabolism in the bilateral basal ganglia, putamen, pons and cerebellum, compared to PD and controls [156, 157]. A multimodal study combining FDG-PET with DTI detected an elevated *D̄* in posterior putamen of MSA-*P* patients that corresponded with local reductions in FDG metabolism [158], suggesting an association between putaminal microstructural damage and related metabolic dysfunction in the brain.

Relative to controls, PSP patients commonly show glucose hypometabolism in the basal ganglia, midbrain, anterior cingulate cortex, frontal lobe and primary motor cortex [153, 157, 159]. Juh et al. compared PSP patients with PD, MSA and controls, and found significant metabolic impairments in the caudate nucleus, thalamus, midbrain, and cingulate gyrus [156]. Thalamic hypometabolism was also a common finding but may not be present in all cases [154, 156]. To distinguish PSP from MSA and CBS, Botha et al. recently proposed the ‘pimple sign’ – an oval/round-shaped region representing midbrain hypometabolism on FDG-PET images. This sign had a high specificity (100%) but low sensitivity (29%) in the ‘definite’ PSP group (PSP-*R*) [160].

^18^F-FDG-PET reveals an asymmetric hypometabolism in the basal ganglia, thalamus and frontoparietal cortical regions among CBS patients, contralateral to the clinically affected side [153, 161]. Niethammer et al. recently utilized spatial covariance analysis to identify a metabolic pattern in clinically-diagnosed CBS patients versus controls [161]. The pattern was characterized by bilateral, asymmetric metabolic reductions including frontoparietal cortex, thalamus, and caudate nucleus, which distinguished CBS from MSA, although not from PSP [161]. Distinction between CBS and PSP was achieved by using asymmetry scores combined with the PSP-related metabolic pattern. Parietal lobe hypometabolism may also help differentiate CBS from PSP and normal controls [159]. Computer-assisted analysis of FDG-PET images (obtained at the initial referral) using Statistical Parametric Mapping (SPM) achieved greater-than 90% concordance with the clinical diagnosis in PS [162], and in some cases proved to be superior to the visual interpretation [157]. Such computer-assisted methods show promise for applications in places where experienced FDG-PET readers are unavailable.

In DLB, hypometabolism in the occipital cortices along with less prominent metabolic decline in the hippocampus was observed relative to AD [163]. Lateral occipital cortex hypometabolism showed the highest sensitivity (88%), whereas the relative preservation of posterior cingulate metabolism (the ‘cingulate island sign’) achieved the highest sensitivity (100%) for diagnosing DLB [164]. Patients with PDD and DLB may exhibit a similar pattern of glucose hypometabolism involving bilateral inferior, medial frontal and right parietal regions, although when compared directly, a more prominent hypometabolism involving the anterior cingulate cortex became evident among the DLB cases [165].

Analysis of resting-state FDG-PET data using spatial covariance method has identified reproducible metabolic patterns in PD and atypical PS. Eidelberg and colleagues identified a PD-related pattern (PDRP) characterized by increased pallido-thalamic and pontine metabolic activity, with relative declines in SMA, premotor cortex, and parietal association regions [166]. PDRP expression showed a linear relationship with motor assessments, and distinguished PD from atypical PS and controls [167]. Similarly, a distinct PD-related cognitive pattern (PDCP) was identified involving metabolic reductions mainly in the medial frontal and parietal association regions, with relative increases in cerebellar cortex and dentate nuclei [166]. PDCP expression correlated with memory and executive performances in PD, and appeared unaltered by routine antiparkinsonian treatment [168]. Specific patterns of abnormal metabolic activity have also been elucidated in CBS [161], MSA and PSP patients [169]. Relative to controls, the MSA-related pattern was identified by metabolic declines in the putamen and cerebellum; whereas, the PSP-related pattern was characterized by decreased metabolism in the brainstem and medial frontal cortex [166, 169].

**Table:** 

**Clinical viewpoint** In patients with parkinsonism presenting with atypical features (see list immediately below), we recommend as a minimum that structural imaging with high resolution brain MRI be pursued, including volumetric T1, T2/FLAIR, gradient-recalled echo and/or SWI sequences. This will allow for visualization of regional atrophy patterns and neuroimaging signatures seen in some atypical parkinsonian disorders, and exclude structural lesions such as tumours and vascular pathology (e.g., strokes, white matter hyperintensities, microbleeds). In complex cases, perfusion SPECT or FDG-PET, as well as DAT-SPECT may be considered to help sort out the differential diagnosis. Atypical features - Poor response to at least 900 mg total daily dose of levodopa - Rapidly progressive course of parkinsonism - Early falls - Early dysphagia - Other neurological signs (e.g., upper motoneuron findings, cerebellar features, supranuclear gaze palsy) - Early dysautonomia - Early prominent cognitive impairment or dementia - Early prominent behavioural changes - Early prominent language changes - Apraxia - Early psychotic features	

### Amyloid imaging

Amyloid PET commonly shows greater cortical Aβ deposition in AD patients relative to DLB, PDD, PD and normal controls. In 80% of DLB patients, an elevated ^11^C-PIB uptake was observed in cortical association areas, cingulum and striatal regions versus controls, while normal uptake was detected in 80% of PDD and all PD subjects [170]. Similarly, a greater mean precuneus ^11^C-PIB uptake was detected in DLB patients versus PD, PD-MCI and PDD that correlated with cognitive decline [171]. Although, most studies find greater ^11^C-PIB cortical retention in DLB versus PD/PDD patients, some studies have not found noteworthy differences [172], which may be due to variability in the underlying pathology. When PD and PDD patients were contrasted, most studies revealed no differences in ^11^C-PIB binding; however, when subjects were reclassified as Aβ-positive and Aβ-negative based on a study-defined threshold, the PDD group contained a greater proportion of Aβ-positive subjects indicating the potential contribution of amyloid pathology to cognitive decline in PDD [173]. Possession of at least one Apolipoprotein E (*APOE*) ɛ4 allele was associated with greater ^11^C-PIB retention in DLB, PDD and PD-MCI [171].

The presence of Aβ pathology in DLB may influence the timing of dementia onset relative to motor symptoms, the severity of cognitive impairment, as well as dementia progression [171, 172]. Neuropathological studies confirm these findings by demonstrating greater amyloid pathology in DLB versus PDD/PD [174] and in PDD versus PD [175]. Claassen et al. observed glucose hypometabolism in regions corresponding to Aβ deposition in DLB, whereas amyloid abnormalities were virtually absent in MSA [176]. Another amyloid radiotracer with a high affinity for Aβ, ^18^F-florbetaben, produced results comparable to ^11^C-PIB and re-confirmed greater neocortical Aβ loads in AD patients relative to PD, DLB and normal controls [177].

### Tau imaging

Spurred by the success of ^11^C-PIB imaging in quantifying Aβ loads, recent efforts have been directed towards developing novel probes to reliably estimate tau accumulation in human brains. Notable challenges of this technique include: 1) the intracellular nature of most tau aggregates, 2) multiple conformations of tau isoforms in the brain, and 3) higher Aβ brain concentrations relative to tau. Other tracer-specific challenges include: 1) the ideal tracer lipophilicity to achieve adequate tracer permeability and clearance, 2) the need for greater selectivity relative to Aβ especially in AD brains, and 3) faster kinetics to reduce toxicity and facilitate timely clearance [161]. Several novel fluorine-18 labelled ligands have been developed that include: 2-(1-(6-[(2-[^18^F]fluoroethyl)(methyl)amino]-2-naphthyl)ethylidene)malononitrile (^18^F-FDDNP); quinoline and arylquinoline derived radiotracers, such as 2-(4-aminophenyl)-6-(2-[^18^F]fluoroethoxy)quinoline (^18^F-THK523) and 6-[(3-[^18^F]fluoro-2-hydroxy)propoxy]-2-(4-dimethyl-aminophenyl)quinolone (^18^F-THK5105); 7-(6-fluoropyridin-3-yl)-5H-pyrido [4,3-b]indole (^18^F-T807, known as ^18^F-AV-1451); as well as ^11^C-labelled phenyl/pyridinyl-butadienyl-benzothiazoles/benzothiazolium derivative (^11^C-PBB3) [178]. Quantifying and determining the topological distribution of tau is crucial to further understand the progression of tauopathies *in vivo*, to clarify the role of neurofibrillary tangles in AD along with their Aβ plaques associations, as well as to improve the sensitivity and specificity of diagnosing PSP and CBD.

Kepe et al. applied ^18^F-FDDNP PET in 15 patients with PSP and detected tracer retention in regions known to be involved in PSP pathology [179]. Subcortical uptake was observed in striatum, thalamus, subthalamus, midbrain and cerebellar white matter regions (versus PD), and high midbrain and subthalamic uptake distinguished PSP patients from PD and controls [179]. ^11^C-PBB3 tracer was shown by Maruyama et al. to bind to tau inclusions in PSP and CBD postmortem brain tissues [180].

Recently, ^18^F-AV-1451 was reported by Marquié et al. to bind tau lesions composed primarily of paired helical filaments in AD brains, without significant selectivity for straight tau filaments in 4-repeat tauopathies, or to lesions containing β-amyloid, α-synuclein, or TDP-43 aggregates [181, 182]. An off-target binding to NM-containing neurons in the midbrain was also identified [182, 183]. This was pursued by Hansen et al., demonstrating a visually apparent decline in ^18^F-AV-1451 signal in the midbrain of PD patients versus controls [183]. Although, nigral ^18^F-AV-1451 signal showed no correlation with the disease duration, motor dysfunction, or striatal ^123^I-FP-CIT values, its utility as an *in vivo* marker of NM-containing cells in the SN was supported. Lower ^18^F-AV-1451 binding in SN in PD was confirmed by another study, including lack of correlation with motor severity in both PD and PSP [184]. Relatively greater (but non-significant) ^18^F-AV-1451 signal was detected in putamen, globus pallidus, subthalamic and dentate nucleus in PSP versus controls, along with variable non-specific subcortical binding in controls [184]. Similarly, no statistically significant differences in ^18^F-AV-1451 uptakes were found in cortical or subcortical regions in PSP versus PD or controls bringing into question the reliability of this tracer in primary tauopathies, such as PSP (Personal communication, Strafella AP). Smith et al. found elevated ^18^F-AV-1451 retention in the basal ganglia of PSP patients, albeit with extensive overlap and age-dependent increases in both groups [185]. Compared to AD, Whitwell et al. reported a greater ^18^F-AV-1451 signal in cerebellar dentate and pallidum of PSP patients, whereas, signal across the cortex was elevated in AD versus PSP [186]. Future efforts need to be directed towards developing improved tracers as well as to test the usefulness of existing tracers in clinical studies.

### Imaging neuroinflammation

In the context of neurodegenerative diseases, the term 'neuroinflammation' refers to chronic immune response in the central nervous system (CNS), characterized by local cellular and microvasculature changes associated with microglial recruitment and proinflammatory cytokine production. Microglia are the resident macrophages and part of the innate immune response of the CNS, representing approximately 10% of the brain’s cell population. In addition to being important players in neurodevelopment and maturation, microglia are involved in tissue repair, maintenance, and regeneration. Emerging evidence highlights that, depending upon the injury and microenvironmental conditions, microglia-mediated inflammatory processes may aggravate injury and contribute to the cascade of events leading to neurodegeneration [187]. A common PET ligand for imaging neuroinflammation has been 1-(2-chlorophenyl-N-methylpropyl)-3-isoquinolinecarboxamide (PK11195), which binds to 18 kDa mitochondrial translocator protein (TSPO, formally peripheral benzodiazepine receptor). Upregulation of TSPO is indicative of augmented microglial activation in the CNS.

Using ^11^C-(*R*)-PK11195, Gerhard et al. reported increased binding in the pons, basal ganglia, and frontal and cingulate cortices in PD versus controls. However, no correlation of microglial activity with clinical severity or ^18^F-dopa was observed [188]. The extent of microglial activation remained stable in a subsample followed longitudinally for two years [188]. Conversely, Ouchi et al. found elevated binding in the midbrain contralateral to the clinically affected side in PD (versus controls), correlating with putaminal DAT levels (using ^11^C-β-CFT-PET) and motor severity [189]. In PDD, widespread microglial activation compared to controls was found in the anterior/posterior cingulate, striatum as well as in the frontal, temporal, parietal and occipital cortices, with maximal parieto-occipital involvement. The spatial extent of cortical binding was greater in PDD than in PD [190]. Elevated tracer binding in MSA (versus controls) was observed in the prefrontal cortex, putamen, pallidum, pons, and SN, consistent with the known neuropathological distribution [191]. Similarly, increased microglial activity in PSP was demonstrated in the basal ganglia, midbrain, frontal lobe, and cerebellum relative to controls [188]; while in CBS, elevated microglial response was noted in the caudate nucleus, putamen, SN, pons, pre/postcentral gyrus and frontal lobe [192].

Recently, Fan et al. evaluated correlations among microglial activation (using ^11^C-(*R*)-PK11195), glucose metabolism (^18^F-FDG), and amyloid load (^11^C-PIB) in AD, MCI, and PDD patients. An inverse correlation was noted between microglial activation and glucose metabolism in temporo-parietal cortex in AD and PDD; whereas, a positive correlation was apparent between microglial activation and amyloid load in AD and MCI [193].

A new tracer, ^11^C-DPA713, has been reported to have a greater sensitivity for TSPO and may emerge as a better indicator of global microglial activation than ^11^C-(*R*)-PK11195 [194]. Limitations of imaging studies of neuroinflammation are small sample sizes and lack of autopsy-verified diagnosis. A viable application of this technique is in monitoring therapeutic responses in clinical trials.

## [^123^I]metaiodobenzylguanidine myocardial scintigraphy in Parkinsonian disorders

[^123^I]Metaiodobenzylguanidine (^123^I-MIBG) is a radioiodinated analogue of guanethidine that is taken up by the postganglionic adrenergic neurons using cellular mechanisms identical to norepinephrine. Upon depolarization, ^123^I-MIBG is released into the synaptic cleft, like norepinephrine, but remains unmetabolized. This uptake and localization of ^123^I-MIBG provides a useful measure of postganglionic sympathetic fiber integrity and function [195]. ^123^I-MIBG myocardial scintigraphy has traditionally been utilized to assess sympathetic nerve damage in cardiovascular diseases. More recently, its application in the differential diagnosis of neurodegenerative diseases has emerged, especially in α-synucleinopathies where profound cardiovascular dysautonomia can be observed [196]. Uptake of ^123^I-MIBG in myocardial scintigraphy is often reported as a heart-to-mediastinum (H/M) ratio of count densities, whereas washout rate index may also be assessed using early and delayed images.

Impairments in ^123^I-MIBG H/M ratios were found to be independent of the duration and severity of autonomic and parkinsonian symptoms [197, 198], intensity of anti-parkinsonian therapy [199], and striatal denervation as assessed using (+)-^11^C-dihydrotetrabenazine (^11^C-DTBZ) PET in PD [200]. Marked decrease in ^123^I-MIBG uptakes were detected relative to controls, even at the early PD stages in the absence of clinically apparent dysautonomia, indicating this technique as a useful diagnostic tool for PD [201–203]. Patients with PSP, MSA and CBS exhibit normal or mildly reduced uptakes, which may help differentiate PD from atypical PS [200, 204]. Many studies report markedly reduced ^123^I-MIBG H/M ratios in patients with LBD [200, 204, 205], compared to normal ratios in AD and healthy controls [197, 205]. ^123^I-MIBG scintigraphy can distinguish LBD from other dementias with high discriminative accuracy (sensitivity 95% and specificity 87%) [205]. This technique was found to be more sensitive than occipital hypoperfusion using SPECT [206] and superior to CSF Aβ and tau biomarkers, in differentiating DLB from AD [207]. King et al. conducted a pooled analysis of ^123^I-MIBG studies and identified two distinct clusters that were well separated at an optimal H/M ratio of 1.77 [208]. This meta-analysis allowed discrimination between PD and MSA, as well as between the two most common neurodegenerative dementias of AD and DLB at a critical ^123^I-MIBG uptake threshold [208]. Similarly, a meta-analysis of 19 studies using myocardial scintigraphy presented a pooled sensitivity of 88% in detecting PD, and a pooled specificity of 85% in distinguishing PD from other PS [209]. A recent study by Sudmeyer et al. utilized a multimodal approach using ^123^I-FP-CIT, ^123^I-IBZM and ^123^I-MIBG and proposed an algorithm that distinguished atypical PS from PD with 94% sensitivity and specificity [210]. Similarly, Sengoku et al. assessed olfactory bulb and tract volume together with H/M ratio of 1.6 to optimally distinguish PD patients from an atypical PS group, comprised of MSA, PSP and CBS participants [211].

Myocardial scintigraphy using ^123^I-MIBG is a useful technique that can offer valuable adjunctive evidence in clinically uncertain cases. However, there is a need to validate this technique in larger pathologically-confirmed cohorts to account for the confounding effects of mixed and vascular pathologies. Various cardiovascular morbidities, latent cardiac disorder and medications may damage the postganglionic sympathetic neurons leading to false-positive findings. Additionally, ^123^I-MIBG H/M ratios may also decrease with age and show gender-specific variations [212], making it essential to use well-matched subgroups in clinical investigations (Table 3).

## Transcranial sonography

Transcranial sonography (TCS) is a low-cost, non-invasive and widely available B-mode neuroimaging technique that uses ultrasound to assess the echogenicity of brain tissues through the intact cranium. TCS is commonly applied using a 2.5 MHz phased-array transducer placed at the preauricular site across the transtemporal bone window. SN is identified at the mesencephalic plane within the brainstem, which appears as a butterfly-shaped structure of low echogenicity surrounded by hyperechogenic basal cisterns. Visualization of the basal ganglia can be achieved in the plane of the third ventricle. A limitation with TCS is that it may not be feasible in some subjects due to an insufficient bone-window.

### Substantia nigra echogenicity

Increased echogenicity of the SN, often visible as an enlarged ‘patchy’ region near the lateral midbrain, is a characteristic TCS finding in PD [213, 214]. This ‘echofeature’ shows no consistent correlation with the disease duration or severity [213, 215], although one such study did observe this finding [216]. In a five-year follow-up study, Berg at al. established that the area of SN hyperechogenicity remains stable over time in PD patients [217], implicating its role as an early (or possibly preclinical) ‘trait’ marker or feature of PD that is independent of disease duration or severity. Spiegel at al. employed ^123^I-FP-CIT SPECT together with TCS and found no association between presynaptic DAT degeneration and SN hyperechogenicity [218], suggesting independent pathophysiological mechanisms for these two modalities. Notably, healthy subjects with enlarged SN echogenic areas but without any motor deficits exhibited marked decrease in striatal ^15^F-dopa on PET, providing evidence of dysfunction in the SN; however, due to the cross-sectional nature of this study, it is unknown if these subjects went on to develop parkinsonism [219, 220]. Another study showed 57% of PD patients with bilateral and 43% with unilateral SN hyperechogenicity, proposing that the bilateral finding may be a more specific marker of PD [215].

Studies report hyperechogenicity of SN (especially when marked) to be highly sensitive (90-91%) and specific (82-96%) for diagnosing PD versus MSA and PSP subjects [214, 221]. It is important to note that SN hyperechogenicity is not a single specific marker of PD as it may also be observed in about 9% of healthy individuals and 16% of essential tremor patients [219, 222]. This ‘echofeature’ is also observed in DLB, PDD and CBS patients to varying degrees [223, 224]. Walter et al. found all DLB, 97% PDD and 94% PD patients with at least unilateral SN hyperechogenicity; whereas 80% of DLB presented with marked bilateral findings [223]. Other studies report 66-88% of CBS patients with markedly echogenic SN [224, 225], whereas, 0-20% of the PSP patients presented with this feature [224–226]. A recent investigation further compared PSP-*R* and PSP-*P* phenotypes, and found SN hyperechogenicity in 6/7 PSP-*P* patients as compared to 1/27 PSP-*R*, implicating that this characteristic may be specific to PSP-*P* [227]. Walter et al. tried to discriminate DLB from PDD using TCS by combining SN echogenic sizes, asymmetry indices and age of onset, and observed high diagnostic accuracy (sensitivity 96%, specificity 80%) [223]. The etiology of SN hyperechogenicity remains unclear and may reflect higher levels of iron in SN [220]. It is plausible that other factors in addition to iron accumulation may contribute to this characteristic, as other iron-rich regions of the brain, such as the pallidum, appear normal in TCS.

**Table 3:** Common SPECT, PET, myocardial scintigraphy, and transcranial sonography findings, compared to healthy controls

Imaging Modality	Measure		Neurodegenerative Disorders									Relevant References
			Synucleinopathies						Tauopathies			
			Lewy Body Spectrum Disorders									
		HC	PD-*n*	PD-*t*	PD-MCI	PDD	DLB	MSA	PSP	CBS	AD	
SPECT												
ST presynaptic	DAT density	○	↓	↓	↓	↓	↓	↓	↓	↓	○	104-111
ST postsynaptic	D2 receptor density	○	↑	○	×	○ ↓	○ ↓	↓	↓	○ ↓	○	105, 108, 111, 129-131
PET												
ST presynaptic	DAT or AADC activity	○	↓	↓	↓	↓	↓	↓	↓	↓	○	139-145
ST postsynaptic	D2 receptor density	○	○ ↑	○	×	×	↓	↓	↓	↓	○	143, 148-151
Cerebral amyloid	^11^C-PIB uptake	○	○	○	○ ↑	○ ↑	↑	○	○	○ ↑	↑	170-173
Cerebral tau	Tau tracers uptake	○	○	○	○	○ ↑	○ ↑	○	○ or ↑	○ ↑	↑	179-186
Neuroinflammation	Microglial activity	○	↑	↑	↑	↑	↑	↑	↑	↑	↑	188-193
Scintigraphy												
Myocardial	^123^I-MIBG uptake	○	↓	↓	↓	↓	↓	○ ↓	○ ↓	○ ↓	○	201-205
TC Sonography												
Substantia nigra	Echogenicity	○ ↑	↑	↑	×	↑	↑	○ ↑	↑↓	↑↓	○ ↑	213, 214, 223-227
Lentiform nucleus	Echogenicity	○ ↑	↑↓	↑↓	×	↑	↑	↑	↑↓	○ ↑	○ ↑	215, 223, 225, 226

Legend: ○ = normal; ↓ = decrease; ↑ = increase, ↑↓ = increase or decrease or normal; ○ ↑ = predominantly normal with increases also reported; ○ ↓ = predominantly normal with decreases also reported; × = not specifically reported in literature

Abbreviations: *AADC*, aromatic amino acid decarboxylase; *AD*, Alzheimer’s disease; *CBS*, corticobasal syndrome; *D2*, dopamine D2; *DAT*, dopamine transporters; *DLB*, dementia with Lewy bodies; *HC*, healthy controls; *MSA*, Multiple system atrophy; *PDD*, Parkinson’s disease dementia; *PD-MCI*, Parkinson’s disease with mild cognitive impairment; *PD-n*, drug-naïve Parkinson’s disease; *PD-t*, drug-treated Parkinson’s disease; *PSP*, progressive supranuclear palsy; *ST*, Striatal; *TC*, transcranial; ^*11*^ *C-PIB*, ^*11*^C-labelled Pittsburg Compound B; ^*123*^ *I-MIBG*, ^*123*^I-labelled metaiodobenzylguanidine

### Lentiform nucleus echogenicity

Hyperechogenicity of the LN is a feature frequently observed in some atypical PS. Normal SN echogenicity together with hyperechogenic LN strongly supports a diagnosis of atypical PS (positive predictive value, 0.96) [215]. Specifically, this echogenic pattern indicated MSA-*P* or PSP diagnosis with 100% sensitivity, but with a relatively low specificity of 59% [226]. Normal SN echogenicity alone supported a diagnosis of MSA-*P* rather than PD (sensitivity 90%; specificity 98%), whereas LN hyperechogenicity alone may not be helpful in the differential diagnosis of PS as 25-30% of PD and PSP patients also display this feature [226]. Sadowski at al. compared TCS patterns in PSP and CBS patients, and detected normal LN echogenicity in all 11 CBS patients [225]. The authors noted that LN hyperechogenicity with coexisting SN normoechogenicity can help exclude CBS as a potential diagnosis among patients with atypical PS [225]. Walter et al. reported that a combination of hyperechogenic LN with third-ventricle dilation of greater than 10-mm suggests a diagnosis of PSP over PD, with 84% sensitivity and 98% specificity [226].

## Conclusions

Extensive neuropathological overlap and clinical heterogeneity in disease presentation and progression, in part, challenges the differential diagnosis of PD and atypical PS, especially at early stages. Imaging biomarkers can provide supportive evidence to aid in the diagnostic process. Multiple biomarkers derived from different imaging modalities can provide distinct information about neuroanatomical and pathophysiological processes associated with the underlying disease. This ‘multimodal approach’ can assist in making early, objective, and confident diagnostic decisions in clinical as well as research settings. Further research is needed to improve measurement techniques, standardize research protocols, and identify effective and pathology-specific radiotracers. Efforts are also needed to replicate these findings in larger well-characterized cohorts. Future clinical decision-making and personalized treatment regimens are sure to rely upon multimodal *in vivo* imaging, not only to improve the diagnostic accuracy but also to track treatment effectiveness in clinical trials.

## Abbreviations

AADC: Aromatic amino acid decarboxylase
AD: Alzheimer’s disease
ADC: Apparent diffusion coefficient
Aβ: Amyloid-beta
CBD: Corticobasal degeneration
CBS: Corticobasal syndrome
CNS: Central nervous system
Cr: Creatine
*D̄*: Mean diffusivity
DAT: Dopamine transporter
DLB: Dementia with Lewy bodies
DTI: Diffusion tensor imaging
FA: Fractional anisotropy
FLAIR: Fluid-attenuated inversion recovery weighted
FTD: Frontotemporal lobar degeneration
GM: Gray matter
H/M: Heart-to-mediastinum ratio of count densities
LBD: Lewy body spectrum disorder
LN: Lentiform nucleus
MCI: Mild cognitive impairment
MCP: Middle cerebellar peduncle
MRI: Magnetic resonance imaging
MRS: Magnetic resonance spectroscopy
MSA: Multiple system atrophy
MSA-*C*: Multiple system atrophy - cerebellar subtype
MSA-*P*: Multiple system atrophy - parkinsonian subtype
NAA: N-acetyl aspartate
NM: Neuromelanin
PD: Parkinson’s disease
PDCP: Parkinson’s disease related cognitive pattern
PDD: Parkinson’s disease dementia
PDRP: Parkinson’s disease related pattern
PET: Positron emission tomography
PS: Parkinsonian syndromes
PSP: Progressive supranuclear palsy
PSP-*P*: Progressive supranuclear palsy - parkinsonian type
PSP-*R*: Progressive supranuclear palsy - Richardson type
SCP: Superior cerebellar peduncle
SMA: Supplementary motor area
SN: Substantia nigra
SNpc: Substantia nigra pars compacta
SPECT: Single photon emission computed tomography
SW: Susceptibility-weighted
T1: T1-weighted
T2: T2-weighted
TCS: Transcranial sonography
TDP-43: Transactive response DNA binding protein-43
TSPO: Mitochondrial translocator protein
VBM: Voxel-based morphometry
VMAT2: Vesicular monoamine transporter type-2
WM: White matter
